# A micro costing of NHS cancer genetic services

**DOI:** 10.1038/sj.bjc.6602270

**Published:** 2004-12-07

**Authors:** G L Griffith, R Tudor-Edwards, J Gray, R Butler, C Wilkinson, J Turner, B France, P Bennett, (The GenQuest research team)

**Affiliations:** 1 Centre for the Economics of Health, Institute of Medical and Social Care Research, Wheldon Building, University of Wales, Bangor LL57 2UW, UK; 2 Institute of Medical Genetics, Cardiff and Vale NHS Trust, University Hospital of Wales, Heath Park, Cardiff CF14 4XW, UK; 3 North Wales Section, Department of General Practice, University of Wales College of Medicine, Wrexham Technology Park, Wrexham LL13 7YP, UK; 4 Department of Psychology, University of Wales, Swansea, Singleton Park, Swansea SA2 8PP, UK

**Keywords:** genetic, breast, ovarian, colorectal, cost

## Abstract

This paper presents the first full micro costing of a commonly used cancer genetic counselling and testing protocol used in the UK. Costs were estimated for the Cardiff clinic of the Cancer Genetics Service in Wales by issuing a questionnaire to all staff, conducting an audit of clinic rooms and equipment and obtaining gross unit costs from the finance department. A total of 22 distinct event pathways were identified for patients at risk of developing breast, ovarian, breast and ovarian or colorectal cancer. The mean cost per patient were £97–£151 for patients at moderate risk, £975–£3072 for patients at high risk of developing colorectal cancer and £675–£2909 for patients at high risk of developing breast or ovarian cancer. The most expensive element of cancer genetic services was labour. Labour costs were dependent upon the amount of labour, staff grade, number of counsellors used and the proportion of staff time devoted to indirect patient contact. With the growing demand for cancer genetic services and the growing number of national and regional cancer genetic centers, there is a need for the different protocols being used to be thoroughly evaluated in terms of costs and outcomes.

For over a century clinicians have been aware that a hereditary predisposition to develop cancer exists in certain families (Steel *et al*, 1999). In total, 5% of breast cancer cases are believed to be due to inherited genetic mutations (Lynch *et al*, 1984); 10–11.7% of ovarian cancer cases (Landis *et al*, 1999; Risch *et al*, 2001; Malanders *et al*, 2004) are believed to be the result of breast cancer susceptibility one and two (BRCA1/2) mutations (Miki *et al*, 1994; Wooster *et al*, 1995). The hereditary genetic disorders of nonpolyposis colorectal cancer (HNPCC) and familial adenomatous polyposis coli (FAP or FAPC) are believed to be responsible for 2–7 and 1% of colorectal cancer cases, respectively (Soravia *et al*, 1997; Aaltonen *et al*, 1998).

With the availability of commercial genetic testing and the development of regional cancer genetics clinics in England, and national services in Wales and Scotland, physicians can now refer families to ascertain their genetic risk. Despite a burgeoning literature upon the psychosocial impact of familial cancer and accessing genetic services upon patients (Audrain *et al*, 1998; Lerman *et al*, 1998; Brain *et al*, 2000; Clarke *et al*, 2001; Geer *et al*, 2001; Rees *et al*, 2001; Fry *et al*, 2003), full and partial economic evaluations of cancer genetic services are sparse (Griffith *et al*, 2004). In this paper, we present the first full micro costing of a commonly used cancer genetic counselling and testing protocol used in the UK with patients at increased risk of developing breast, ovarian, breast and ovarian (breast ovarian) or colorectal cancer. The micro costing was conducted at the Cardiff clinic of the Cancer Genetics Service in Wales (CGSW) as part of a multimethod evaluation of that service by the GenQuest research team.

Figure 1 illustrates the service provided to patients referred to the CGSW. Primary, secondary and tertiary care clinicians throughout Wales have been issued with referral guidelines to aid them in assessing whether or not a patient should be referred to cancer genetic services (See Table 1). Once a patient is referred, they are issued by post with a general information pack on genetic cancer and a questionnaire asking them about their family history of cancer. If the questionnaire is not returned within 4 weeks, a reminder letter is issued. Upon receipt of a completed family history questionnaire, a risk assessment is carried out. Cyrilic software is used to establish the patient's risk of developing cancer; all patients will either be at high, moderate or population risk. All high and moderate risk patients, with the exception of those at moderate risk of developing breast cancer, then have their family history checked against medical records. Finally, all four counsellors (team leader, service and practice development coordinator and two consultants, see Table 2 for staff) will discuss the patient's family history, confirm the risk status and the most appropriate care for the patient.

In the case of an inappropriate referral, a patient believed to be at increased risk (moderate or high) due to their self-reported family history but subsequently found to be at population risk following examination of medical records and risk assessment, the general practitioner (GP) and referrer are informed. The GP and/or referrer contact the patient, reassure them that they are at population risk and advise them to adhere to general health awareness measures and to attend national screening programmes when they are eligible.

All moderate risk patients are telephoned and informed of their risk status by the team leader or service and practice development coordinator. Patients at moderate risk of developing breast or colorectal cancer are encouraged to discuss any fears and questions they have and receive counselling over the phone at a time that is convenient for them. Patients at risk of developing ovarian or breast ovarian cancer are also contacted by phone but are invited to come to the clinic for a face to face counselling session with a counsellor (team leader, service and practice development coordinator or consultants [MC21/02]). Following counseling, breast, ovarian and breast ovarian patients are referred by the genetics service and colorectal patients by their GP/referrer for presymptomatic care.

All high-risk patients are informed over the phone by a genetic counsellor (team leader, service and practice development coordinator or consultants [MC21/02]) that they are at high risk and are invited to come to the clinic for a counselling session. The preliminary counselling session includes a full discussion of the implications, issues and procedures involved in genetic testing and presymptomatic care. As confronting the issues associated with genetic disease can induce psychological distress among some patients (Lerman *et al*, 1997; Grosfeld *et al*, 2000; Fry *et al*, 2003), they are reminded that they are free to withdraw from genetic testing at any time. For patients who wish to proceed with genetic testing, a living cancer-affected relative is then invited to come to the genetics clinic for counselling and to have a blood sample taken for genetic testing. While the cancer-affected relative's blood is being tested, the presymptomatic patient will give blood and attend counselling twice with a minimum of a 1 month interval between both visits. Two blood samples are taken on separate visits to minimise the possibility of identification errors occurring in relation to blood/DNA samples. A 1 month interval between the counselling sessions is used to allow time for patients to consider the implications of testing and ask further questions. This counselling protocol is derived from the highly successful Huntington's protocol developed at the Institute of Medical Genetics in Cardiff. During counseling, presymptomatic patients are prepared for the possibility that they have an established mutation predisposing them to develop cancer, there is a mutation in their family but they have not inherited it (however, a close relative may have inherited the mutation) or that their family does not have an established mutation but their family history suggests that their family have an as yet unidentified mutation. In the former and latter cases, presymptomatic patients are referred for presymptomatic care. In the penultimate case, patients are reassured that they are at population risk and advised to adhere to general health awareness measures and to attend national screening programmes when they become eligible.

Molecular genetic testing for mutations predisposing patients to develop cancer currently comprises of conducting mutation screening and presymptomatic testing by means of sequence analysis. A screening test is the first test conducted for a high-risk family; a cancer-affected relative is tested for a breast, ovarian, breast ovarian, HNPCC or FAP mutation. In the case of breast and/or ovarian cancer, the Cardiff laboratory conducts full mutation screening of all 22 relevant exons (45 fragments) of the BRCA1 gene and all 26 relevant exons (40 fragments) of the BRCA2 gene for a cancer-affected relative. Exons of the BRCA1 gene are prescreened by Denaturing Gradient Gel electrophoresis (DGGE) and electrophoretic variants are sequenced in both directions to determine the nature of the DNA sequence variant and therefore whether it is responsible for the inherited breast/ovarian cancer in that family (i.e. it is pathogenic). Similarly, exons of the BRCA2 gene are prescreened by Denaturing High Performance Liquid Chromatography (DHPLC); variants are again further assessed by bidirectional sequence analysis. For HNPCC, 19 exons (19 fragments) of the MLH1 and 16 exons (16 fragments) of the MSH2 genes are searched. For FAP, 15 exons (24 fragments) of the APC gene are fully searched. The MLH1, MSH2 and APC genes are screened directly by bidirectional sequence analysis, thus the pathogenicity of variants is directly determined.

Presymptomatic testing (PST) is provided to relatives of a cancer-affected patient that has been found to be a mutation carrier by a mutation screening test. Presymptomatic testing is conducted upon the BRCA1, BRCA2, MLH1, MSH2 and the APC genes by bidirectional sequence analysis of duplicate DNA samples from the at risk relative alongside a control sample from the cancer-affected patient in which the familial pathogenic mutation has previously been identified. In the past, patients suspected of having an APC mutation predisposing them to FAP and with a suitable family structure were identified by means of linkage analysis. Linkage analysis is being phased out in Cardiff but has been included in this paper as it may still be in use in some small regional laboratories.

For information purposes we include below the presymptomatic care that patients are referred for by the cancer genetics service. Presymptomatic care in not costed in this paper, only the capital, labour and overheads used in referring a patient for presymptomatic care are included in the costing. Presymptomatic care for patients at increased (moderate and high) risk of developing cancer includes surveillance and/or prophylactic surgery. Surveillance for women at increased risk of developing breast cancer is provided in the form of annual mammography from the age of 35 (high risk)/40 (moderate risk) to 50 years and every 18 months from 50 to 60 years of age. At 60 years, women enter the national screening programme and receive mammography every 3 years. The alternative to surveillance is to have prophylactic surgery in the form of bilateral prophylactic mastectomy, oophorectomy with mammography or both surgeries. Patients at increased risk of developing colorectal cancer (HNPCC or FAP) receive a combination of surveillance and surgery; colonoscopy every 2 (high risk)/5 (moderate risk) years and polypectomy as required. Surveillance commences at 5 years prior the youngest cancer incidence in the family, with a minimum age of 20 years for high-risk patients and 25 for moderate risk patients. Alternatively, patients can opt for the prophylactic surgery options of subtotal colectomy or protocolectomy. Women at increased risk of developing ovarian cancer have the surgical option of having prophylactic oophorectomy. Women at increased risk that are referred to the CGSW can have presymptomatic surveillance despite the fact that there is currently no evidence-based surveillance strategy for them. Surveillance is provided as part of the UK Familial Ovarian Cancer Screening Study (UKFOCSS) (Jacobs *et al*, 2000), which is seeking to develop an optimised screening procedure for these women. Women opting to participate in the UKFOCSS study are asked to attend a joint consultation between the genetics service and a consultant surgeon (all patients at increased risk can (colorectal will) see a surgeon and/or oncologist as part of their presymptomatic care but a genetic counsellor will not be present). This involves a 10–15 min consultation for moderate risk women and 10–30 min consultation for those at high risk. Surveillance is in the form of annual ultrasound of the ovaries and CA125 blood testing from the age of 35 years. All costs associated with the UKFOCSS research project have been excluded from the costs that follow in Tables 5 and 9. Women at increased risk of developing breast ovarian cancer are offered the same presymptomatic care as women at increased risk of breast and women at increased risk of ovarian cancer.

## MATERIALS AND METHODS

Establishing from the provider's perspective, the cost per patient of providing cancer genetic services comprised of four main steps:

1. Identifying all event pathways that have resource or clinical outcomes for patients.
2. Measurement of the resources required for each event pathway.
3. Establish the cost per unit of resource.
4. Apply the cost of the resources used to each event pathway.

All event pathways and all of the distinct stages associated with the pathways were identified from service protocols and consultation with the team leader and senior consultant at the clinic. In 2002, the clinic had seven members of clinical and administrative staff and a further three laboratory staff (see Table 2). Labour capital and overhead resources were measured by means of a questionnaire administered to all members of staff and conducting an audit of the clinic rooms and laboratory. Clinical and administrative staff were asked to report on average how long a procedure took them in minutes, the typical quantity of consumables used, to list any equipment used and where each task was undertaken. Laboratory staff were asked what elements of molecular genetic testing they conducted, which equipment was used, which consumables were used and to estimate any wastage, for example spillage etc.

The finance department of the Cardiff and Vale NHS trust was asked to provide costs for staff time, capital, overheads, equipment and consumables. Staff time was valued at actual gross wage rates including National Insurance and pension contributions adjusted for holiday and sick leave. Capital charges for buildings were taken from the annual value assigned by the district valuer. Consumables and equipment were valued at replacement cost.

Having ascertained which steps of an event pathway each member of staff participated in, which rooms were used (including 50% of communal facilities shared with the remainder of the Medical Genetics Service in Wales) and resources required, this information was combined with the information gained from an audit of the furniture and equipment in each room of the clinic to produce an inventory of the rooms and equipment associated with each task. Equipment costs were transformed into an annual cost assuming a 6% discount rate, a 5 year working life and payment in arrears. In addition to annual equipment costs, each room was allocated in terms of total area, a proportion of the annual capital charge, rates, maintenance costs, cleaning, and power and sewerage charges. Laboratory rooms also had the costs associated with laboratory refuse and maintenance of specialist equipment. Total room costs were then calculated for the number of hours annually that they are allocated to the cancer genetics service allowing for holiday and sick leave. As rooms that were not used 100% of the time by the cancer genetics team were used for the remainder of the time by other genetics services such as Huntington's disease fixed costs were fully accounted for.

Using the Committee for Medical Genetics Workload Units Working Group report (2002), each task undertaken by a member of the laboratory staff was transformed into work load units (WLUs). Essentially, a workload unit represents the laboratory and administrative operations completed in a minute (including indirect time such as clinical governance and professional development). As WLUs are applicable to all laboratories in the UK, they have been calculated based upon the minimum batch size and technology representative of small laboratories; however, they can be modified to account for the use of semiautomated technology which is generally employed when screening large genes for unknown mutations. An allowance for medium throughput technology was applied in this study. Based on laboratory records it was possible to ascertain the annual WLUs completed by all laboratory staff in the last 12 months and calculate gross labour costs per WLU and hour.

During administration of the questionnaire to staff it emerged that in addition to the direct time spent in contact with patients and processing their referrals, a wide range of other commitments which recur but are sporadic in terms of timing, and contribute to the workload of staff were cited by clinical, laboratory and administrative staff. These included:

Administrative

- Meeting with surgeons to discuss the appropriateness of their referrals.
- Audit, clinical governance and administrative requests.
- Attendance at trust meetings.
- Team management.

Education and development

- Formal teaching and contributing to conferences.
- Personal education and skills development.
- Facilitating and participating in research.

Estimates of the amount of time that went on indirect work varied between 25 and 75%. To take these commitments into account, 33% of work time was assumed to be devoted to indirect patient contact and labour costs for clinical and administrative staff were inflated accordingly. It was not necessary to do this for the laboratory staff as WLUs already contain an inflation to take account of these additional time commitments.

Annual stationary, photocopying, postage and telephone charges plus courses and training costs were transformed to a cost per hour per WLU based on the total number of hours per WLUs per year each member of staff using each resource worked. Costs are presented in 2002/2003 pounds. As capital and overhead costs were measured in 2001/2002, these costs were inflated to 2002/2003 levels using the Hospital and Community Pay and Price Index (Netten and Curtis, 2003).

## RESULTS

**Figure 1:**
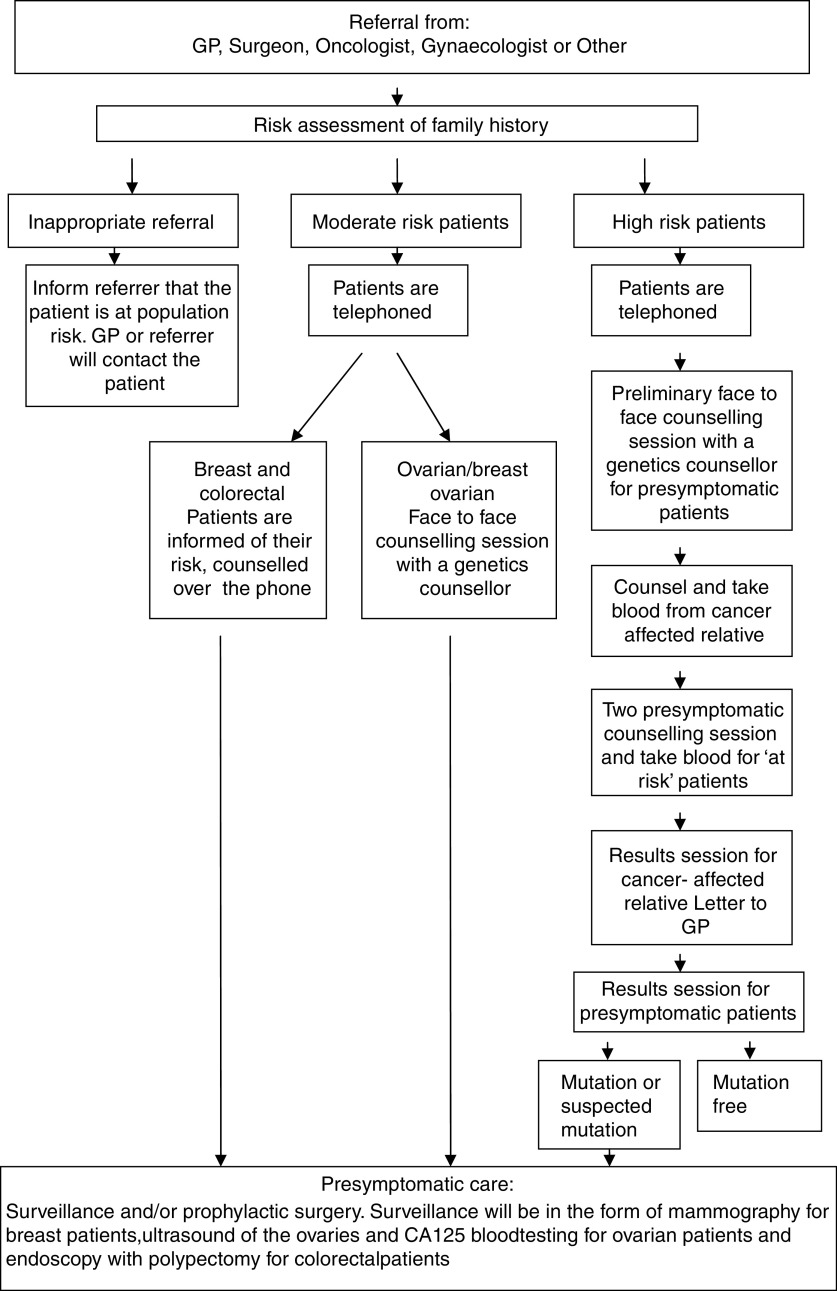
Overview of care provided to patients.

A total of 22 distinct event pathways were identified in this study (see Table 3). Event pathway one represents a patient referred to the cancer genetics service but deciding not to proceed with the referral by not returning the family history questionnaire. The patient will have been issued with a reminder letter 4 weeks after they were issued with the questionnaire. Event pathway two represents an inappropriate referral; a patient believed to be at increased risk due to their self-reported family history but subsequently found to be at population risk following examination of medical records and risk assessment (left hand column of Figure 1). Event pathways three to five are the moderate risk event pathways (centre column of Figure 1). Event pathways six to 22 are the high-risk event pathways (right hand column of Figure 1).

Event pathways 6, 10, 15 and 19 represent high-risk families approaching the cancer genetics service for the first time. Having tested and found a known mutation, for example, mutated BRCA2 or MLH1, the first presymptomatic family member to approach the service (usually the patient originally referred to the genetics service) is tested.

Event pathways 7, 11, 16 and 20 represent families where the cancer-affected relative is tested and no known mutation is found. The presymptomatic patient and his/her first-degree relatives (e.g. sister, mother or daughter) are still at high risk due to their family history. It is likely that these families have mutations that have yet to be identified by genetic scientists. The presymptomatic patients in these event pathways are dealt with in exactly the same way as patients with a known mutation such as MLH1 in their family, except they do not receive genetic testing.

Event pathways 8, 13, 17 and 21 represent the second and any subsequent presymptomatic family members of a family where a cancer-affected relative has been found to have a known mutation. These event pathways only contain the costs associated with testing, counselling and referring for presymptomatic care each presymptomatic patient deciding to approach the cancer genetics service. Event pathway 12 is the forerunner of event pathway 13 and is becoming obsolete. In this event pathway linkage testing is used rather than sequence analysis to identify an APC mutation. Linkage testing is a less sensitive method of testing and requires a suitable family structure.

Event pathways 9, 14, 18 and 22 represent the second and subsequent family members of a high-risk family where no known mutation was found when testing a cancer-affected relative. Presymptomatic patients in this event pathway are dealt with in exactly the same way as patients from families with a known mutation, except that they do not receive genetic testing.

The number of event pathways, number of possible staff combinations within each pathway and the number of distinct steps in each pathway prohibit displaying all the clinical and administrative labour associated with each pathway. Table 4 is an example of the clinical and administrative resources required for event pathway 5, moderate risk – ovarian or breast ovarian cancer. Clinical and administrative consumables are not listed as they were all part of the overhead cost, for example, stationary (all sources of capital and overhead costs are listed in the Materials and Methods section).

In Table 5, we present the clinical and administrative costs of the cancer genetics service in terms of labour, capital and overhead costs (all consumables were included in the overhead costs). Labour formed the majority of the cost for each event pathway. In the case of a patient who does not return their family history questionnaire (event pathway 1), 61% of the mean cost was labour, 23% capital and 16% overheads. The division for an inappropriate referral and the moderate risk event pathways (event pathways 2–5) was 68–70% labour, 17–18% capital and 12–13% overhead. In all, 70–72% of the clinical and administrative cost of high-risk breast cancer event pathways (event pathways 15–18) was labour, 17–19% capital and 11% overheads. For the ovarian/breast ovarian and colorectal event pathways (6–9, 10–14 and 19–22), labour costs amounted to 75–78%, capital 13–15% and overheads 10%.

Tables 6 and 7 present the tasks, resources and consumables required for each molecular testing strategy. In Table 8, the cost of these strategies is presented in terms of capital, overhead and consumable costs. As was the case for clinical and administrative costs, the largest component of the costs was labour. In all, 59–63% of all presymptomatic tests (Sequence analysis and linkage analysis) were labour costs, 8–13% consumables, 20% capital and 9% overheads. A total of 53% of mutation screening for breast and breast ovarian mutations was labour, 13% consumables, 23% capital and 11% overheads. While the largest element of mutation screening for a HNPCC or FAP mutation was labour costs, it amounted to a substantially lower percentage than seen for the other mutation screening tests at 38%. Laboratory consumables costs formed a large element of mutations screening costs for HNPCC and FAP at 28%, 15% higher than for any other test; capital and overhead costs, respectively, amounted to 23 and 11% of the total cost.

Table 9 contains the minimum costs, maximum costs and their mean for all the breast, ovarian/breast ovarian and colorectal cancer risk categories dealt with by the CGSW. An initial referral where the patient decides not to proceed and does not return their family history questionnaire (event pathway 1) resulted in a mean cost of £16. For a patient at moderate risk of breast or colorectal cancer or an inappropriate referral (event pathways 2, 3 and 4), the mean cost was £97–£98. The cost of dealing with a woman at moderate risk of developing ovarian or breast ovarian cancer was the highest of all the moderate risk pathways at £151.

The cost of counselling, testing and referring the first high-risk presymptomatic member of a family and a caner-affected relative (event pathways 6, 10, 15 and 19) for breast, ovarian/breast ovarian or colorectal cancer (HNPCC or FAP) ranged between £2510 and £3072. In the event of no established mutation such as BRCA1 being found when a cancer-affected relative is tested, the cost of testing the cancer-affected relative, counselling and referring the cancer-affected relative and the presymptomatic relative (event pathways 7, 11, 16 and 20) ranged between £1665 and £2039. The cost of genetically testing, counselling and referring any members of a family where a mutation had previously been found (event pathways 8, 12, 13, 17 and 21), for example, a brother or sister and a cancer-affected relative had been through event pathways 6, 10, 15 or 19, ranged between £1,152 and £1,589. The cost of counselling and referring subsequent members of a family where testing had revealed that there was no known mutation such as BRCA2 but their family history placed them at high risk ranged between £675 and £975 (event pathways 9, 14, 18 and 22).

### Sensitivity

To assess the sensitivity of the total cost of each event pathway to the amount of time devoted by clinical and administrative staff to indirect patient contact, the minimum and maximum reported percentages of indirect work time, 25 and 75% were substituted into the labour costs (see final and penultimate columns of Table 9). Given that the largest element of the cost of each event pathway was labour (see Tables 5 and 8), it is not surprising to find that the total cost per event pathway was sensitive to change in the percentage of indirect working time. Assuming 25% of working time to be indirect rather than the 33% used in the base case resulted in costs declined for all event pathways to 87–95% of the means base case costs, for example, Event pathway 1, £13.47/£15.54^*^100=87%. Assuming 75% of work time to be devoted to indirect patient contact resulted in costs rising to 167–267% of the base case, for example, Event pathway 1, £41.44/£15.54^*^100=267%. The event pathways with no laboratory testing, event pathways 1–5, 9, 14, 18 and 22, were most sensitive to change in the percentage of work time devoted to indirect patient contact.

## DISCUSSION

The variation between minimum and maximum costs within each event pathway (Table 9) is a result of the grade of the staff that can be involved and the variation in the time it could take to conduct the work associated with each referral. The differences in the costs of referrals at moderate risk of breast, ovarian and colorectal cancer are a result of the variation in the services and work associated with each type of referral. Usually it is not necessary to obtain medical records for relatives of patients believed to be at moderate risk of developing breast cancer, while this is a necessity for ovarian/breast ovarian and colorectal referrals. The cost of dealing with patients believed to be at moderate risk of ovarian/breast ovarian cancer is greater than that for colorectal cancer. This is a result of colorectal patients receiving phone counselling for 7.5–15.0 min, while ovarian patients attend the genetics clinic for a face to face counselling session of approximately 1-h duration. The face to face counselling and additional counselling time devoted to women at moderate risk of developing ovarian cancer is due to the greater uncertainty surrounding these women. While these women can be positively identified as being at increased risk, there is currently no evidence-based surveillance strategy that can significantly enhance detection and survival chances for them.

An inappropriate referral is as expensive as a referral for a patient at moderate risk of developing breast or colorectal cancer. This is a result of obtaining medical records to check the family history reported by the patient. On average, more patient records are checked for this group of patients and as a result more labour costs are incurred. The discrepancy between actual and reported family history usually stems from a patient misidentifying the location of cancer for a deceased relative or a relative they are no longer in contact with.

As is the case with moderate risk patients, variation in the mean costs associated with referrals for patients at high risk is a result of variation in the procedures involved with each cancer type. A single counsellor deals with patients at high risk of breast cancer. Patients at risk of developing ovarian or colorectal cancer receive all of their counselling sessions with two counsellors, a consultant (MC21/02 or MC21/04) and the team leader (NPU/04) or the service and practice development coordinator (NP51/05), and a cancer-affected relative will receive one of their two counselling sessions with two counsellors. Two counsellors are used due to the complexity of the issues to be addressed and to aid the training of counsellors. In addition, the laboratory costs differ for all molecular genetic tests with the exception of presymptomatic screening (sequence analysis) for breast and/or ovarian mutations (BRCA1 and BRCA2) and HNPCC mutations (MLH1 and MSH2).

Sensitivity analysis revealed that the event pathways with no laboratory testing were most sensitive to change in the percentage of work time devoted to indirect patient contact. This was a result of labour costs contributing less to the laboratory costs than to clinical and administrative costs as can be seen from Tables 5 and 8.

In interpreting the results of this study readers should bear in mind the following factors. Firstly, the measurement of labour for clinical and administrative tasks was based upon the stated responses of seven members of staff to an administered questionnaire. Obviously the more time consuming and expensive option of gathering revealed stochastic data by means of a time and motion exercise on all 22 event pathways for a large cohort of patients would yield incontrovertible data. It would also be preferable to conduct such a study at multiple sites using the same clinical and laboratory protocols, allowing differences by cancer genetic centre to be allowed for. Secondly, due to the small sample size in this study, 10 members of staff, it was not possible to conduct statistical analysis upon the cost estimates. Thirdly, the costs in this study are based upon a service working at full capacity, which is the case for the Cardiff clinic of the CGSW since its inception in 1999. In the unlikely event that demand for cancer genetic services declines (Ponder, 1999), the cost per patient using each event pathway would obviously rise in accordance with the decline in demand. Fourthly, all mutation screening in this study was conducted using medium throughput technology (see Table 6). Smaller laboratories that do not have access to such labour saving devises would obviously incur greater labour and capital costs per test than the Cardiff laboratory. Conversely, a laboratory with more medium throughput devises, for examlple, for presymptomatic tests or technological developments reducing labour input, would result in a reduction in labour costs. Despite the limitations noted above, we believe that the cost estimates derived in this study are representative of the protocol used and are generalisable to similar services and settings.

Lerman (1997) has questioned the appropriateness of counselling protocols such as the one in this study as they are derived from Huntington's disease protocols. Protocols with extensive pretest assessment were initially designed to identify depression and suicidal potential. Brown and Kessler (1995, 1996) and Lerman have called for economic analysis in this field to identify the most cost-effective method of delivering cancer genetic services, including a comparison of delivering cancer genetic services by different clinicians and in different settings. We are wholeheartedly in support of a comprehensive assessment of all competing protocols in terms of costs, health outcomes and patient utility in terms of health outcomes, nonhealth outcomes and process. The health outcomes and utility results gathered in parallel with this costing will be published in detail elsewhere.

In conclusion, the cost of providing cancer genetic services to patients at increased risk of developing breast, ovarian, breast ovarian or colorectal cancer (HNPCC or FAP) are substantial, particularly in the case of high-risk presymptomatic patients from families approaching cancer genetic services for the first time at £2,510–£3,072 (event pathways 6, 10, 15 and 19). The most expensive element of all 22 event pathways was labour costs. Labour costs were dependent upon the grade of staff used to conduct a task, how quickly the task was performed, was there a need to conduct tasks such as obtain medical records and check them, the number of counsellors used (two counsellors were used to counsel high-risk ovarian/breast ovarian and colorectal cancer patients) and the proportion of staff time devoted to indirect patient contact such as audit, clinical governance and administrative requests. With the growing demand for cancer genetic services and the growing number of national and regional cancer genetic centers, there is a need for the protocols being used to be thoroughly evaluated in terms of costs and outcomes. We present this manuscript as a first step in the economic evaluation of alternative contemporary methods of delivering cancer genetic services.

## Figures and Tables

**Table 1:** Referral guidelines

**Cancer**	**Family history criteria (on the same side of the family)**
Breast cancer	• One first-degree relative diagnosed at 40 years or less.
	• Two first-degree relatives diagnosed at 60 years or less.
	• Three first- or second-degree relatives diagnosed at any age.
	• One first-degree male breast cancer.
	• A first-degree relative with bilateral breast cancer.
	
Breast/ovarian cancer	• At least one breast and one ovarian cancer in first-degree relatives (breast cancer diagnosed under 50 years if only one of each cancer).
	• A first-degree relative who has both breast and ovarian cancer.
	
Ovarian cancer	• Two or more ovarian cancers (at least one first-degree relative).
	
Colon cancer	• One first-degree relative diagnosed at 40 years or less.
	• Two first-degree relatives diagnosed at 60 years or less.
	• Three relatives diagnosed at any age (at least one first-degree relative).
	• Familial adenomatous polyposis in a first- or second-degree relative.
	• Hereditary nonpolyposis colorectal cancer (revised Amsterdam criteria) in a first- or second-degree relative.

**Table 2:** Staff and grades

**Title**	**Grade**	**Proportion of time working for the service**	**Gross wages per hour**
Administrative assistant	CR31/05	1.0	£11.19
Administrative assistant	CR31/03	0.07	£10.32
Consultant	MC21/04	1.0	£51.86
Consultant	MC21/02	0.6	£45.46
Coordinator	CR31/03	0.4	£10.32
Team leader	NPU/04	1.0	£22.10
Genetic technologist	MT01	0.1	£8.97
Genetic technologist	MT02	1.0	£10.54
Principal clinical scientist	B-grade 17	1.0	£19.96
Service and practice development coordinator	NP51/05	0.8	£20.41

**Table 3:** Event pathways

1. Family history questionnaire not returned
2. Inappropriate referral
3. Moderate risk – breast cancer
4. Moderate risk – colorectal cancer
5. Moderate risk – ovarian or breast ovarian cancer

High risk – colorectal (HNPCC)

6. Test cancer-affected relative (mutation found) and test presymptomatic patient
7. Test cancer-affected relative (no mutation found) and do not test presymptomatic patient
8. Test subsequent presymptomatic members of a family with an established mutation
9. Counsel and arrange presymptomatic care for members of a family with no established mutation

High risk – colorectal (FAP)

10. Test cancer-affected relative (mutation found) and test presymptomatic patient
11. Test cancer-affected relative (no mutation found) and do not test presymptomatic patient
12. Test subsequent presymptomatic members of a family with an established mutation (using linkage testing)
13. Test subsequent presymptomatic members of a family with an established mutation (using sequence analysis)
14. Counsel and arrange presymptomatic care for members of a family with no established mutation

High risk – breast (BRCA1/2)

15. Test cancer-affected relative (mutation found) and test presymptomatic patient
16. Test cancer-affected relative (no mutation found) and do not test presymptomatic patient
17. Test subsequent presymptomatic members of a family with an established mutation
18. Counsel and arrange presymptomatic care for members of a family with no established mutation

High risk – ovarian or breast ovarian (BRCA1/2)

19. Test cancer-affected relative (mutation found) and test presymptomatic patient
20. Test cancer-affected relative (no mutation found) and do not test presymptomatic patient
21. Test subsequent presymptomatic members of a family with an established mutation
22. Counsel and arrange presymptomatic care for members of a family with no established mutation

HNPCC=hereditary nonpolyposis colorectal cancer; FAP or FAPC=familial adenomatous polyposis coli.

**Table 4:** Tasks undertaken by clinical and administrative staff for patients at moderate risk of ovarian or breast ovarian cancer

	**Task**	**Time (min)**	**Staff**	**Resources**
1.	Record receipt of referral letter	1.0	Administrative assistant (CR31/05)	Office
2.	Input client details to database and issue family history questionnaire	3.5	Administrative assistant (CR31/05)	Office
3.	Record return of family history and update database	2.0	Administrative assistant (CR31/05)	Office
4.	Input family history into computer	3.0–7.5	Team leader (NPUV/04)	Office
5.	Obtain permission to access relatives' medical records	3.0–7.5	Team leader (NPUV/04)	Office
6.	Obtain records	3.0	Coordinator (CR31/03)	Office
7.	Check medical records	4.0–7.5	Team leader (NPUV/04)	Office
8.	Review family history	4.0–15.0	Team leader (NPUV/04) and service and practice development coordinator (NP51/05) and consultant in cancer genetics (MC21/02) and consultant in cancer genetics (MC21/04)	Offices and Seminar room
10.	Letter and booklet to patient and letter to referrer	10.0	Administrative assistant (CR31/03)	Office
11.	Counselling session	50.0–60.0	Team leader (NPUV/04) or service and practice development coordinator (NP51/05) or consultant in cancer genetics (MC21/02)	Offices and counselling room
12.	Refer to Breast Test Wales and/or gynaecologist/surgeon if appropriate	10.0	Team leader (NPUV/04) or service and practice development coordinator (NP51/05) or consultant in cancer genetics (MC21/02)	Office
13.	Fill in database	5.0	Coordinator (CR31/03)	Office
	Communal facilities	64–97.5^a^		

Communal facilities include phlebotomy room (take blood), waiting room, toilets and hallways.

a Total time devoted to a single patient by one or more members of staff minus overlap with other patients. There are no laboratory inputs for this event pathway.

**Table 5:** Counselling and administrative costs

	**Counselling and administrative costs**			
**Event pathway**	**Labour (min**–**max)**	**Capital (min**–**max)**	**Overheads (min**–**max)**	**Total (min**–**max)**
1. Family history questionnaire not returned	£9 (£9–£10)	£4 (£2–£5)	£2 (£2–£3)	£16 (£14–£17)
2. Inappropriate referral	£69 (£37–£100)	£17 (£8–£26)	£12 (£6–£18)	£98 (£51–£144)
3. Moderate risk – breast cancer	£66 (£27–£105)	£18 (£8–£28)	£13 (£6–£20)	£97 (£40–£153)
4. Moderate risk – colorectal cancer	£69 (£37–£100)	£17 (£8–£26)	£12 (£6–£18)	£98 (£51–£144)
5. Moderate risk – ovarian or breast ovarian cancer	£105 (£51–£158)	£27 (£17–£37)	£20 (£11–£28)	£151 (£79–£223)
				
High risk – colorectal (HNPCC)				
6. Test cancer-affected relative (mutation found) and test presymptomatic patient	£1068 (£593–£1542)	£188 (£111–£265)	£139 (£99–£180)	£1396 (£803–£1988)
7. Test cancer-affected relative (no mutation found) and do not test presymptomatic patient	£622 (£338–£906)	£119 (£70–£167)	£85 (£58–£113)	£826 (£466–£1186)
8. Test subsequent presymptomatic members of a family with an established mutation (MLH1/ MSH2 mutation in family)	£777 (£432–£1121)	£125 (£77–£173)	£95 (£71–£120)	£997 (£579–£1415)
9. Counsel and arrange presymptomatic care for members of a family with no established mutation	£759 (£415–£1103)	£122 (£74–£171)	£93 (£68–£118)	£975 (£557–£1392)
				
High risk – colorectal (FAP)				
10. Test cancer affected relative (mutation found) and test presymptomatic patient	£1068 (£593–£1542)	£188 (£111–£265)	£139 (£99–£180)	£1396 (£803–£1988)
11. Test cancer-affected relative (no mutation found) and do not test presymptomatic patient	£622 (£338–£906)	£119 (£70–£167)	£85 (£58–£113)	£826 (£466–£1186)
12. Test subsequent presymptomatic members of a family with an established mutation (using linkage testing)	£777 (£432–£1121)	£125 (£77–£173)	£95 (£71–£120)	£997 (£579–£1415)
13. Test subsequent presymptomatic members of a family with an established mutation (using sequence analysis)	£777 (£432–£1121)	£125 (£77–£173)	£95 (£71–£120)	£997 (£579–£1415)
14. Counsel and arrange presymptomatic care for members of a family with no established mutation	£759 (£415–£1103)	£122 (£74–£171)	£93 (£68–£118)	£975 (£557–£1392)
				
High risk – breast (BRCA1/2)				
15. Test cancer-affected relative (mutation found) and test presymptomatic patient	£705 (£264–£1146)	£181 (£106–£256)	£110 (£67–£153)	£996 (£437–£1555)
16. Test cancer-affected relative (no mutation found) and do not test presymptomatic patient	£430 (£173–£687)	£115 (£68–£162)	£70 (£42–£98)	£614 (£283–£946)
17. Test subsequent presymptomatic members of a family with an established mutation	£496 (£170–£822)	£119 (£73–£166)	£72 (£45–£99)	£688 (£288–£1087)
18. Counsel and arrange presymptomatic care for members of a family with no established mutation	£487 (£165–£809)	£117 (£70–£164)	£71 (£44–£98)	£675 (£279–£1070)
				
High risk – ovarian or breast ovarian (BRCA1/2)				
19. Test cancer-affected relative (mutation found) and test presymptomatic patient	£1068 (£593–£1542)	£188 (£111–£265)	£139 (££99–£180)	£1396 (£803–£1988)
20. Test cancer-affected relative (no mutation found) and do not test presymptomatic patient	£622 (£338–£906)	£119 (£70–£167)	£85 (£58–£113)	£826 (£466–£1186)
21. Test subsequent presymptomatic members of a family with an established mutation	£777 (£432–£1121)	£125 (£77–£173)	£95 (£71–£120)	£997 (£579–£1415)
22. Counsel and arrange presymptomatic care for members of a family with no established mutation	£759 (£415–£1103)	£122 (£74–£171)	£93 (£68–£118)	£975 (£557–£1392)

Costs are rounded to the nearest £1 within each column. HNPCC=hereditary nonpolyposis colorectal cancer; FAP or FAPC=familial adenomatous polyposis coli.

**Table 6:** Workload units (WLUs) per molecular genetic test

**Cancer and mutations**	**Type of analysis**	**Molecular strategy**	**WLU**	**Staff**	**Resources**
Breast, ovarian or breast ovarian	Mutation screening	• Sample reception/DNA extraction	24	MT01	Lab
BRCA1/2		*BRCA1:*			
		• DGGE for exons 2–24 (45 fragments using MT^a^ technology)	506	MT02	Lab
		• Rearrangement analysis (dosage PCR)	120	MT02	Lab
		• Sequence analysis of variants	320	B-grade 17	Lab and 0ffice
		• Report	15	B-grade 17	
		*BRCA2:*			
		• DHPLC for exons 2–27 (40 fragments using MT^a^ technology)	450	MT02	Lab
		• Sequence analysis of variants	320	B-grade 17	Lab and office
		• Report	15	B-grade 17	
					
	Presymptomatic testing	• Sample reception/DNA extraction (duplicate samples)	48	MT01	Lab
		Sequence analysis of known mutation	440	B-grade 17	Lab and office
		• Report	15	B-grade 17	
HNPCC	Mutation screening	• Sample reception/DNA extraction	24	MT01	Lab
*MLH1/MSH2*		*MLH1:*			
		• Sequence analysis (19 fragments using MT^a^ technology)	1520	MT02	Lab
		*MSH2:*			
		• Sequence analysis (16 fragments using MT^a^ technology)	1280	MT02	Lab
		• Report	15	B-grade 17	Lab and office
	Presymptomatic testing	• Sample reception/DNA extraction (duplicate samples)	48	MT01	Lab
		• Sequence analysis of known mutation	440	B-grade 17	Lab and office
		• Report	15	B-grade 17	
					
					
FAP	Mutation screening	• Sample reception/DNA extraction	24	MT01	Lab
APC		• APC sequence analysis (24 fragments using MT^a^ technology)	1920	MT02	Lab
		• Report	15	B-grade 17	Lab and office
	Presymptomatic testing	• Sample reception/DNA extraction (duplicate samples)	48	MT01	Lab
		• Sequence analysis of known mutation	440	B-grade 17	Lab and office
		• Report	15	B-grade 17	
	Presymptomatic testing	• Sample reception/DNA extraction (duplicate samples plus family samples)	144	MT01	Lab
	(Linkage analysis)	• Polymorphic marker analysis (six samples for two markers)	540	B-grade 17	Lab and office
		• Report	60	B-grade 17	

a Weighted WLUs for the use of medium throughput (MT) technology. HNPCC=hereditary nonpolyposis colorectal cancer; FAP or FAPC=familial adenomatous polyposis coli.

**Table 7:** Molecular genetic testing consumables and unit costs

**Method**	**Components (supplier)**	**Cost per unit (£)**
DNA extraction	Whatman kit (Abbott Diagnostics, Maidenhead, Berkshire, UK)	£3.50
Polymerase chain reaction (PCR)	• 166 mM (NH_4_)2SO_4_, 670 mM Tris-HCl (pH 8.3), 37 mM M MgCl_2_, 850 *μ*g/ml BSA	£0.66
	• 0.5 *μ*M forward and reverse primers (synthetic DNA molecules – Invitrogen, Paisley, Scotland, UK)	
	• 0.5 U *Taq* polymerase (Invitrogen, Paisley, Scotland, UK)	
	• 750 *μ*M deoxyribonucleotides (Invitrogen, Paisley, Scotland, UK)	
	• 100 ng genomic DNA	
Gel electrophoresis	• DGGE (9% (37.1 acyrlamide : 1 bis-acrylamide) polyacrylamide, 0–100% formamide, 1 × TAE)	£0.24
	• DHPLC (Transgenomics, Crewe, Cheshire, UK)	
	• Automated DNA sequencer (Applied Biosystems, Foster City, CA, USA)	
Sequence analysis	• ABI BigDye3.0 kit (Applied Biosystems, Foster City, CA, USA)	£3.19
Licence fee	• Payable for use of patented PCR technology (Roche Diagnostics, Lewes, East Sussex, UK)	£2.70 (single test) £5.60 (multiple tests)

Costs exclude value added tax.

**Table 8:** Laboratory costs per molecular genetic test

**Test**	**Consumables (extract blood sample)**	**Laboratory consumables**	**Labour**	**Capital**	**Overheads**	**Total**
Breast, ovarian or breast ovarian – Mutation screening	£0.19	£138.67	£560.76	£239.76	£110.48	£1049.86
Breast, ovarian or breast ovarian – Presymptomatic testing	£0.38	£59.63	£271.58	£90.96	£41.42	£463.97
HNPCC – Mutation screening	£0.19	£338.46	£464.38	£278.81	£130.33	£1212.17
HNPCC – Presymptomatic testing	£0.38	£59.63	£271.58	£90.96	£41.42	£463.97
FAP – Mutation screening	£0.19	£235.45	£323.58	£192.16	£89.81	£841.19
FAP – Presymptomatic testing	£0.38	£46.06	£271.58	£90.96	£41.42	£450.40
FAP – Presymptomatic testing (Linkage analysis)	£0.38	£46.06	£371.04	£120.14	£54.69	£592.31

All consumables include VAT. HNPCC=hereditary nonpolyposis colorectal cancer; FAP or FAPC=familial adenomatous polyposis coli.

**Table 9:** Total cost per event pathway

				**Sensitivity**	
**Event pathway**	**Counselling and administrative costs (min**–**max)**	**Laboratory costs**	**Total cost (min**–**max)**	**25% of time on indirect work (min**–**max)**	**75% of time on indirect work (min**–**max)**
1. Family history questionnaire not returned	£16 (£14–£17)	£0.00	£16 (£14–£17)	£13 (£12)	£41 (£36–£47)
2. Inappropriate referral	£98 (£51–£144)	£0.00	£98 (£51–£144)	£85 (£44–£125)	£260 (£137–£384)
3. Moderate risk – breast cancer	£97 (£40–£153)	£0.00	£97 (£40–£153)	£84 (£35–£133)	£258 (£107–£408)
4. Moderate risk – colorectal cancer	£98 (£51–£144)	£0.00	£98 (£51–£144)	£85 (£44–£125	£260 (£137–£384)
5. Moderate Risk – ovarian or breast ovarian cancer^a^	£151 (£79–£223)	£0.00	£151 (£79–£223)	£131 (£69–£194)	£400 (£208–£592)
					
High risk – colorectal (HNPCC)					
6. Test cancer-affected relative (mutation found) and test presymptomatic patient^b^	£1396 (£803–£1988)	£1676	£3072 (£2479–£3664)	£2887.79 (£2,373.76–£3401.82)	£5371.49 (£3799.73–£6943.25)
7. Test cancer-affected relative (no mutation found) and do not test presymptomatic patient^b^	£826 (£466–£1186)	£1213	£2039 (£1678–£2399)	£1929.86 (£1617.39–£2242.32)	£3397.61 (£2442.01–£4353.24)
8. Test subsequent presymptomatic members of a family with an established mutation (MLH1/ MSH2 mutation in family)	£997 (£579–£1415)	£464	£1461 (£1043–£1879)	£1329.32 (£966.80–£1691.86)	£3104.66 (£1996.40–£4212.92)
9. Counsel and arrange presymptomatic care for members of a family with no established mutation	£975 (£557–£1392)	£0.00	£975 (£557–£1392)	£846 (£484–£1208)	£2581 (£1475–£3688)
					
High risk – colorectal (FAP)					
10. Test cancer-affected relative (mutation found) and test presymptomatic patient^b^	£1396 (£803–£1988)	£1292	£2687 (£2095–£3280)	£2503 (£1989–£3017)	£4987 (£3415–£6559)
11. Test cancer-affected relative (no mutation found) and do not test presymptomatic patient^b^	£826 (£466–£1186)	£842	£1668 (£1307–£2028)	£1558.88 (£1246.41–£1871.34)	£3026.63 (£2071.03–£3982.26)
12. Test subsequent presymptomatic members of a family with an established mutation (using linkage testing)	£997 (£579–£1415)	£592	£1589 (£1171–£2007)	£1458 (£1095–£1820)	£3233 (£2125–£4341)
13. Test subsequent presymptomatic members of a family with an established mutation (using sequence analysis)	£997 (£579–£1415)	£450	£1447 (£1029–£1865)	£1316 (£953–£1678)	£3091 (£1983–£4199)
14. Counsel and arrange presymptomatic care for members of a family with no established mutation	£975 (£557–£1392)	£0.00	£975 (£557–£1392)	£846 (£484–£1208)	£2,581 (Ł1475–£3688)
					
High risk – breast (BRCA1/2)					
15. Test cancer-affected relative (mutation found) and test presymptomatic patient^b^	£996 (£437–£1555)	£1514	£2510 (£1950–£3069)	£2379 (£1894–£2864)	£4143 (£2660–£5626)
16. Test cancer-affected relative (no mutation found) and do not test presymptomatic patient^b^	£614 (£283–£946)	£1050	£1665 (£1333–£1996)	£1584 (£1296–£1872)	£2671 (£1791–£3551)
17. Test subsequent presymptomatic members of a family with an established mutation	£688 (£288–£1087)	£464	£1152 (£752–£1551)	£1061 (£715–£1408)	£2280 (£1221–£3340)
18. Counsel and arrange presymptomatic care for members of a family with no established mutation	£675 (£279–£1070)	£0.00	£675 (£279–£1070)	£586 (£243–£929)	£1782 (£733–£2831)
					
High risk – ovarian or breast ovarian (BRCA1/2)^c^					
19. Test cancer-affected relative (mutation found) and test presymptomatic patient^b^	£1396 (£803–£1988)	£1514	£2909 (£2317–£3502)	£2725 (£2211–£3240)	£5209 (£3637–£6781)
20. Test cancer-affected relative (no mutation found) and do not test presymptomatic patient^b^	£826 (£466–£1186)	£1050	£1876 (£1516–£2236)	£1768 (£1455–£2080)	£3235 (£2280–£4191)
21. Test subsequent presymptomatic members of a family with an established mutation	£997 (£579–£1415)	£464	£1461 (£1043–£1879)	£1329 (£967–£1692)	£3105 (£1996–£4213)
22. Counsel and arrange presymptomatic care for members of a family with no established mutation	£975 (£557–£1392)	£0.00	£975 (£557–£1392)	£846 (£484–£1208)	£2581 (£1475–£3688)

Costs are rounded to the nearest £1 within each column. HNPCC=hereditary nonpolyposis colorectal cancer; FAP or FAPC=familial adenomatous polyposis coli.

a Joint ovarian clinic for moderate risk ovarian/breast ovarian: min =£27.26, max =£51.44, mean = £39.35.

b If the cancer-affected relative of a high-risk patient is too ill to attend the cancer genetics clinic, a genetic counsellor will make a home visit. A home visit would result in a mean net increase of £36.60 to the cost of testing a cancer-affected relative and the presymptomatic patient.

c Joint ovarian clinic for high-risk ovarian/breast ovarian: min =£36.75, max =£110.25, mean = £73.50.

## References

[bib1] Aaltonen LA, Salovaara R, Kristo P, Canzian F, Hemminki A, Peltomak P, Chadwick RB, Kaariainen H, Eskelinen M, Jarvinen H, Mecklin JP, de la Chapelle A, Percesepe A, Ahtola H, Harkonen N, Julkunen R, Kangas E, Ojala S, Tulikoura J, Valkamo E (1998) Incidence of hereditary nonpolyposis cancer and the feasibility of molecular screening for the disease. N Engl J Med 338(21): 1481–1487 9593786 10.1056/NEJM199805213382101

[bib2] Audrain J, Schwarts MD, Lerman C, Hughes C, Peshkin BN, Biesecker B (1998) Psychological distress in women seeking genetic counselling for breast ovarian cancer risk: the contributions of personality and appraisal. Ann Behav Med 19: 370–377 10.1007/BF02895156 9706364

[bib3] Brain K, Gray J, Norman P, Parsons E Clarke A, Rogers C, Mansel R, Harper P (2000) Why do women attend familial breast cancer clinics? J Med Genet 37: 1–5 10699056 10.1136/jmg.37.3.197 PMC1734549

[bib4] Brown ML, Kessler LG (1995) The use of gene tests to detect hereditary predisposition to cancer: Economic considerations. J Natl Cancer Inst 87: 1131–1136 7674317 10.1093/jnci/87.15.1131

[bib5] Brown ML, Kessler LG (1996) Use of gene tests to detect hereditary predisposition to cancer: What do we know about cost-effectiveness? Int J Cancer 69: 55–57 8600063 10.1002/(SICI)1097-0215(19960220)69:1<55::AID-IJC14>3.0.CO;2-J

[bib6] Clarke S, Bluman LG, Borstelmann N, Regan K, Winer EP, Rimer BK, Skinner CS (2001) Patient motivation, satisfaction and coping in genetic counseling and testing for BRCA1 and BRCA2. Journal of Genetic Counseling 9: 219–235 10.1023/A:1009463905057 26141318

[bib7] Committee for Medical Genetics Workload Units Working Group (2002) CMGS Workload Units: Proposed Scheme. The Committee for Medical Genetics

[bib8] Fry A, Cull A, Appleton S, Rush R, Holloway S, Gorman D, Cetnarskyj R, Thomas R, Campbnell J, Anderson E, Steel M, Porteous M, Campbell H (2003) A randomised control treial of breast cancer genetics services in South East Scotland: Psychologicxal impact. Br J Cancer 89: 653–659 12915873 10.1038/sj.bjc.6601170 PMC2376929

[bib9] Geer KP, Ropka ME, Cohn WF, Jones SM, Miesfeldt S (2001) Factors influencing patients' decisions to decline cancer genetic counselling services. Journal of Genetic Counseling 10: 25–40 11767799 10.1023/a:1009451213035

[bib10] Griffith GL, Edwards RT, Gray J (2004) Cancer Genetics Services: A Review of the Economic Evidence and Issues. Br J Cancer 90: 1697–1703 15150621 10.1038/sj.bjc.6601792 PMC2410279

[bib11] Grosfeld FJM, Lips CJM, Beemer FA, ten Kroode HFJ (2000) Who is at risk for psychological distress in genetic testing programs for hereditary cancer disorders? Journal of Genetic Counseling 9(3): 253–266 26141320 10.1023/A:1009468005966

[bib12] Jacobs I, Mackay J, Menon U, Skates S (2000) UK Familial Ovarian Cancer Screening Study: Study Protocol. Cancer Research UK and University College London Cancer Trials Centre http://www.ncrn.org.uk/portfolio/data.asp?ID=1069

[bib13] Landis SH, Murray T, Bolden S, Wingo PA (1999) Cancer statistics. CA Cancer J Clin 49: 8–31 10200775 10.3322/canjclin.49.1.8

[bib14] Lerman C (1997) Translational behavioural research in cancer genetics. Preventative Medicine 26: S65–S69 10.1006/pmed.1997.0204 9327494

[bib15] Lerman C, Hughes C, Lemon SJ, Main D, Snyder C, Durham C, Norad S, Lynch HT (1998) What you don't know can hurt you: adverse psychological effects in members of BRCA1-linked and BRCA2-linked families who declined genetic testing. J Clin Oncol 16: 1650–1654 9586874 10.1200/JCO.1998.16.5.1650

[bib16] Lerman C, Schwartz MD, Lin TH, Hughes C, Norad S, Lynch HT (1997) The influence of psychological distress on use of genetic testing for cancer risk. J Consult Clin Psychol 65(3): 414–420 9170764 10.1037//0022-006x.65.3.414

[bib17] Lynch HT, Albano WA, Danes BS, Layton MA, Kimberling WJ, Lynch JF, Cheng SC, Costello KA, Mulcahy GM, Wagner CA, Tindall SL (1984) Genetic predisposition to breast cancer. Cancer 53: 612–622 6581856 10.1002/1097-0142(19840201)53:3+<612::aid-cncr2820531306>3.0.co;2-5

[bib18] Malanders S, Ridderheim M, Masback A, Loman N, Kristoffersson U, Olsson H, Nibert M, Boirg A (2004) One in 10 ovarian cancer patients carry germline BRCA1 or BRCA2 mutations. Results of a prospective study in Southern Sweden. Eur J Cancer 40(3): 422–428 14746861 10.1016/j.ejca.2003.09.016

[bib19] Miki Y, Swensen J, Shattuck-Eidens D, Futreal PA, Harshman K, Tavtigian S, Liu Q, Cochran C, Bennett LM, Ding W, Bell R, Rosenthal J, Hussey C, Tran T, McClure M, Frye C, Hattier T, Phelps R, Haugen-Strano A, Katcher H, Yakumo K, Gholami Z, Shaffer D, Stone S, Bayer S, Wray C, Bogden R, Dayananth P, Ward J, Tonnin P, Narod S, Bristow PK, Norris FH, Helvering L, Morrison P, Rosteck P, Lai M, Barrett JC, Lewis C, Neuhausen S, Cannon-Albright L, Goldgar D, Wiseman R, Kamb A, Skolnick MH (1994) A strong candidate for the breast and ovarian cancer susceptibility gene BRCA1. Science 266: 66–71 7545954 10.1126/science.7545954

[bib20] Netten A, Curtis L (2003) Unit Costs of Health and Social Care. Canterbury: Personal Social Services Research Unit, University of Kent at Canterbury

[bib21] Ponder BAJ (1999) Costs, benefits and limitations of genetic testing for cancer risk. Br J Cancer 80(Supplement 1): 46–50 10466762

[bib22] Rees G, Fry A, Cul A (2001) A family history of breast cancer: women's experiences from a theoretical perspective. Soc Sci Med 52: 1433–1440 11286366 10.1016/s0277-9536(00)00248-3

[bib23] Risch HA, McLaughlin JR, Cole DEC, Rosen B, Bradley L, Kwan E, Jack E, Vesprini DJ, Kuperstein G, Abrahamson JLA, Fan I, Wong B, Narod SA (2001) Prevalence and penetrance of germline BRCA1 and BRCA2 mutations in population series of 649 women with ovarian cancer. Am J Hum Genet 68: 700–711 11179017 10.1086/318787 PMC1274482

[bib24] Soravia C, Bapat B, Cohen Z (1997) Familial adenomatous polyposis (FAP) and hereditary nonpolyposis colorectal cancer (HNPCC): a review of clinical, genetic and therapeutic aspects. Schweiz Med Wochenschr 127: 682–690 9140167

[bib25] Steel M, Smyth E, Vasen H, Eccles D, Evans G, Moller P, Hodgson S, Stoppa-Lyonnet D, Chang-Claude J, Caligo M, Morrison P, Haites N (1999) Ethical, social and economic issues in familial breast cancer: A compilation of views from the E.C. Biomed II Demonstration project. Dis Markers 15: 125–131 10595265 10.1155/1999/564893 PMC3851615

[bib26] Wooster R, Bignell G, Lancaster J, Swift S, Seal S, Mangion J, Collins N, Gregory S, Gumbs C, Micklem G (1995) Identification of the breast cancer susceptibility gene BRCA2. Nature 378: 789–792 8524414 10.1038/378789a0

